# Progressive Medicine

**Published:** 1904-07

**Authors:** 


### BOOK REVIEWS.

---

Progressive Medicine. Vol. VI. No. 2, June 11, 1904, Lea Bros. & Co., Philadelphia and New York. Six Dollars per Annum.

The present volume of Progressive Medicine is devoted to surgery of the abdomen, gynecology, diseases of the blood, diathetic and metabolic diseases, diseases of the spleen, thyroid gland and lymphatic system, ophthalmology. The contributors are Wm. B. Coley, John G. Clark, Alfred Stengel and Edward Jackson. The volume is well illustrated and fully indexed.

---

It is reported by a contemporary that a woman of Boston, eighty years old, and worth $75,000, is hiding herself because she says physicians want her body to dissect, and all for the reason that her floating ribs have by muscular action been transferred from her right side to her left side. She says that recently when she was in a hospital for treatment her malformation was discovered, and she was offered $2,000 on condition that she would bequeath her body for dissection. She fears that the doctors won't be content to wait until she dies, and so she has concealed herself. Her malformation is said to be unique, in that it has been caused by gradual muscular action.

